# Disproportionate preponderance of HPV genotypes associated with anogenital warts among HIV-positive MSM

**DOI:** 10.3389/fpubh.2024.1437309

**Published:** 2024-09-20

**Authors:** Wegene Borena, Maria Kitchen, Martin Gisinger, Ninon Taylor, Hannes Oberkofler, Diyani Dewasurendra, Andreas Widschwendter, Heribert Stoiber, Dorothee von Laer, Mario Sarcletti

**Affiliations:** ^1^Institute of Virology, Medical University of Innsbruck, Innsbruck, Austria; ^2^Department of Dermatology and Venerology, Medical University of Innsbruck, Innsbruck, Austria; ^3^Department of Internal Medicine III, Paracelsus Medical University, Salzburg, Austria; ^4^Dr. Ninon Taylor’s Practice for Internal Medicine and HIV Medicine, Salzburg, Austria; ^5^Department of Laboratory Medicine, Paracelsus Medical University, Salzburg, Austria; ^6^Manson Unit, Médecins sans Frontières, London, United Kingdom; ^7^Department of Obstetrics and Gynecology, Medical University of Innsbruck, Innsbruck, Austria

**Keywords:** HIV-positive MSM, HPV vaccine, high-risk HPV, low-risk HPV, gay men, anogenital warts, anal HPV infection

## Abstract

**Background:**

In this study, we characterized the HPV genotype distribution in a population of 489 adults already positive for HPV DNA. The study population was divided into two groups: 244 HIV-positive (HIV+) men who have sex with men (MSM) undergoing routine anal screening for sexually transmitted diseases (STDs) and 245 women undergoing routine cervical cancer screening. Acknowledging the fact that women and MSM represent two independent circles of sexual practices, which are—largely—exclusive of each other, we were interested in determining if particular genotypes of human papillomavirus (HPV) disproportionately predominate in one of these circles compared to the other.

**Results:**

HIV+ MSM are significantly more likely to be infected with multiple genotypes at a time, with an odds ratio (OR) of 9.30 (95% confidence interval [CI]: 3.91–22.1) and a *p*-value of <0.001. In addition, multivariable-adjusted logistic regression analysis showed that anal swab samples were significantly more likely to harbor lrHPV infections, with an OR of 6.67 (95% CI: 2.42–18.4) and a *p*-value of <0.001, in particular, HPV 6, with an OR of 8.92 (95% CI: 3.84–20.7) compared to cervical samples of screening women.

**Conclusion:**

Given the significant impact of recurrent anogenital warts (AGWs) on quality of life and the accompanying predisposition to invasive anal cancer, our data underscore the critical need for HPV vaccination. This includes expanding vaccination eligibility to include both boys and adults within high-risk populations.

## 1 Introduction

HPV is one of the most common sexually transmitted pathogens. Over 200 HPV genotypes exist, categorized into high-risk (hrHPV) and low-risk (lrHPV) types based on the potential to cause malignant or merely benign lesions (1–6). Most HPV infections are transient in nature, resulting in no or low-grade lesions that often regress spontaneously. In a few cases, however, some hrHPV infections may persist and lead to severe malignant lesions (7, 8).

The substantial disease burden caused by HPV infections is not limited to women. Men, particularly MSM, have an especially high risk of anal HPV infection and a high incidence of anorectal carcinoma (9–12). The estimated global cervical HPV prevalence among healthy women is approximately 12%, whereas the prevalence is almost universal among those MSM who are HIV-infected (13–16).

For the prevention of HPV-associated malignancies and AGWs, vaccines have been developed and approved for use since 2006 (17–19). Prevention of HPV infection among MSM needs an immunization program that explicitly targets this population since the expected herd immunity effect following the female-only vaccination program is destined to reach only men who have sex with women (MSW). This understanding that women and MSM represent two independent circles of sexual practices, which are—to a large extent—exclusive of each other, may legitimately raise the suspicion that there could be some peculiarities or differences in the details of HPV genotype distribution between these two groups.

If confirmed that particular genotypes circulate preferentially and predominantly among a particular population group or show a predilection to a particular anatomical site, this may have significant clinical and public health relevance.

For this purpose, we examined and compared the distribution of hrHPV and lrHPV genotypes among population adults already positive for HPV DNA: (1) HIV+ MSM undergoing regular anal swab sampling as part of a routine screening program for STDs and (2) healthy women undergoing routine cervical sampling as part of routine cervical cancer screening program.

## 2 Materials and methods

### 2.1 Study population

As shown in Supplementary Figure 1, the source population consisted of 1,320 healthy women with cervical samples and 286 HIV+ MSM with anal swab samples. In total, 489 HPV-positive individuals [HIV+ MSM (*n* = 244) and healthy women (*n* = 245)] were included in the final analyses. The HPV tests were conducted between March 2013 and October 2017.

### 2.2 HPV-positive MSM

This study population was a sub-cohort of the Austrian HIV Cohort Study (AHIVCOS) living in Vorarlberg, Tirol, and Salzburg and receiving follow-up and antiretroviral therapy at the Medical University of Innsbruck, Department of Dermatology and Venereology and at the University Clinic of Salzburg, Department of Internal Medicine (20). Data on sociodemographic and reproductive/sexual behavior were obtained using questionnaires.

Anal-swab samples were taken (self-collected) as part of a screening program for *Chlamydia trachomatis* (CT) and *Neisseria gonorrhoeae* (NG) infections using *Abbott multi-Collect Specimen Collection Kit* (Abbott, Chicago, IL, USA) that contains approximately 1.2 mL of DNA stabilizing transport. CT/NG detection took place within 3–4 days after sample collection in an automated manner by which an aliquot of approximately 0.5 mL of the sample was transferred to a secondary plate. The remaining (residual) original material was processed for the detection and genotyping of HPV by the latest 1 week after CT/NG testing. HPV testing was conducted between May 2015 and October 2016. Out of a total of 286 men tested for HPV, we selected 244 individuals who were positive for one or more of the HPV genotypes.

### 2.3 HPV-positive healthy screening women

This study population is selected out of 1,320 women aged 18–65 years who underwent cervical swab sampling (physician collected) for cervical cancer screening using cytology. Furthermore, using structured questionnaires, consenting women also filled out questionnaires on sociodemographic, reproductive, and sexual behavior. For the purpose of HPV DNA detection, a second sample was collected using an Abbott Cervi-Collect specimen collection kit (Abbott, Chicago, IL, USA). For this study, we selected all women who tested positive for one or more of the HPV genotypes (*n* = 245).

### 2.4 HPV detection and genotyping

In both study populations, HPV DNA was detected using a sensitive and validated nucleic acid detection and genotyping kit (Ampliquality Type Express, AB ANALITICA, Padua, Italy). After amplification of the L1 genome, genotyping followed using allele-specific reverse line blot hybridization of the PCR product(s) permitting the differentiation of 14 high-risk HPV (hrHPV) (16, 18, 31, 33, 35, 39, 45, 51, 52, 56, 58, 59, 66, and 68a/b) and 26 low-risk HPV (lrHPV) genotypes consisted types (6, 11, 26, 40, 42, 43, 44, 53, 54, 55, 61, 62, 64, 67, 69, 70, 71, 72, 73, 81, 82, 83, 84, 87, 89, and 90). The kit is equipped with a system that controls the quality of sample content and nucleic acid extraction method (beta-globin detection as internal control) as well as positive and negative controls.

### 2.5 Statistical analysis

The genotype distribution pattern of hrHPV and lrHPV was analyzed and stratified by population group. Crude and multivariable-adjusted logistic regression models were used to compute ORs with corresponding 95% confidence intervals (95% CI) for HPV prevalence across the type of study population and several other sociodemographic (age, educational level, marital status) and behavioral (smoking status, number of lifetime sexual partners (LSPs), and age at first sexual contact) parameters. We also conducted a sensitivity analysis, in which we did our comparison in a sub-group of the study population by excluding those with (i) multigenotype infection and (ii) reporting LSP > 10 in an effort to make the two populations comparable. *p*-values of <0.05 were considered significant. We used the statistical program SPSS (IBM Corp., Armonk, NY, USA) for the analysis.

## 3 Results

Examining the source populations, the overall prevalence of HPV was 85.3% (95% CI: 80.7–89.0) among HIV+ MSM (244 out of 286 HPV-tested individuals) and 18.6% (95% CI: 16.6–20.8) among screening women (245 out of 1,320 HPV tested women) (Supplementary Figure 2). Owing to the high overall prevalence of HPV, the probability of being positive for multiple, high-risk, low-risk, and selected HPV genotypes is generally high among MSM, putting them at a higher risk of developing any type of HPV-associated lesions.

The rest of the analysis concentrated on characterizing the distribution of HPV genotypes only among participants (*n* = 489) who tested positive for any HPV DNA.

### 3.1 Baseline characteristics

Table 1 presents details of sociodemographic and reproductive/sexual behavior across the study population. The mean age (SD) of the study participants was 37.5 (13.2) years, whereby MSM were significantly older [mean age (SD) 44.8 (12.6) vs. 30.3 (9.3)]. With an almost 20 times higher likelihood of having more than 10 LSPs and a significantly lower likelihood of living in a partnership, our study affirms the high-risk sexual behavior among HIV+ MSM compared to women. No statistically significant difference was observed for educational or smoking status or age at first sexual contact.

**Table 1:** Baseline characteristics of HPV-positive study participants [HIV+ MSMs (*n* = 244) and HIV- women† (*n* = 111)].

Variable		Crude OR (95% CI)	Adjusted‡ OR (95% CI)	*p*-value§
Age, years [mean (SD)]				
Healthy women	**30.3 (9.3)**	1		<0.001
HIV+ MSM	**44.8 (12.6)**	1.12 (1.09–1.15)		
Educational status beyond high school, n (%) ^§^				
Healthy women	**61 (55.5)**	1	1	
HIV+ MSM	**62 (41.6)**	0.99 (0.91–1.08)	0.94 (0.85–1.04)	0.21
Married/living in partnership, n (%)^§^				
Healthy women	**71 (65.1)**	1	1	
HIV+ MSM	**90 (41.9)**	**0.39 (0.24–0.62)**	**0.44 (0.26–0.76)**	**0.03**
Current smokers, n (%)^§^				
Healthy women	**62 (56.4)**	1	1	
HIV+ MSM	**115 (50.9)**	1.23 (0.79–1.97)	1.42 (0.84–2.41)	0.19
BMI ≥ 25, n (%)^§^				
Healthy women	**21 (19.4)**	1	1	
HIV+ MSM	**84 (40.0)**	**2.76 (1.59–4.79)**	**3.27 (1.75–6.14)**	**<0.001**
Age at first sexual contact, years (mean (SD)) ^§^				
Healthy women	**16.2 (1.7)**	1	1	
HIV+ MSM	**16.1 (3.3)**	0.99 (0.91–1.08)	0.94 (0.85–1.04)	0.21
Lifetime sexual partners ≥ 10, n (%)^§^				
Healthy women	**10 (10.3)**	1	1	
HIV+ MSM	**107 (6.09)**	**19.3 (9.27–40.6)**	**19.8 (8.80–44.4)**	**<0.001**

SD, standard deviation; IQR, interquartile range; BMI, body mass index (Kg/m^2^); OR, odds ratio; 95% CI, 95% confidence interval. Bold values mean statistically significant.

MSM, Men having sex with men.

†Sociodemographic variables except age are available only for *n* = 111 women.

‡Variables other than “Age” are adjusted for age.

§ *p*-value for adjusted OR.

### 3.2 HPV genotype distribution

Table 2 characterizes HPV genotype distribution across the two study populations. MSM were significantly more likely to harbor multiple HPV genotypes than healthy women even after adjusting for a number of variables, including age, age at first sexual contact, partnership status, and the number of LSPs. The mean number (95% CI) of genotypes per sample was 3.84 (3.52–4.18) among MSM, compared to 2.04 (1.87–2.22) among women, with a significant difference (*p* < 0.001).

**Table 2:** Characterizing genotype distribution pattern among HPV-positive HIV+ MSMs (*n* = 244) with HIV- women (*n* = 245).

Variable		Crude OR (95% CI)	Adjusted† OR (95% CI)	*p*-value‡
Multiple HPV types, n (%)				
Healthy women	130 (53.1)	1	1	
HIV+ MSM	200 (82.3)	**4.11 (2.72–6.23)**	**9.30 (3.91–22.1)**	**<0.001**
HR-HPV, n (%)				
HIV- women	209 (85.7)	1	1	
HIV+ MSM	189 (78.1)	**0.32 (0.15–0.71)**	**0.23 (0.07–0.84)**	**0.03**
LR-HPV, n (%)				
HIV- women	102 (41.6)	1	1	
HIV+ MSM	207 (85.5)	**8.29 (5.35–12.9)**	**6.67 (2.42–18.4)**	**<0.001**
ONLY-HR-HPV, n (%)				
HIV- women	136 (56.2)	1	1	
HIV+ MSM	35 (14.5)	**0.13 (0.09–0.20)**	**0.13 (0.05–0.38)**	**0.001**
ONLY-LR-HPV				
HIV- women	36 (14.9)	1	1	
HIV+ MSM	67 (27.7)	**2.19 (1.39–3.44)**	**4.00 (1.34–12.0)**	**0.013**
Vaccine-type HPV§				
HIV- women	73 (65.8)	1	1	
HIV+ MSM	152 (67.0)	1.42 (0.96–2.10)	1.29 (0.76–2.20)	0.34
HPV 16, n (%)				
HIV- women	74 (30.2)	1	1	
HIV+ MSM	72 (29.6)	0.97 (0.66–1.43)	0.98 (0.59–1.61)	0.92
HPV 18, n (%)				
HIV- women	21 (8.6)	1	1	
HIV+ MSM	26 (10.7)	1.29 (0.70–2.34)	1.20 (0.56–2.56)	0.64
HPV 6, n (%)				
HIV- women	9 (3.7)	1	1	
HIV+ MSM	56 (23.0)	**7.85 (3.79–16.3)**	**8.92 (3.84–20.7)**	<0.001
HPV 11, n (%)				
HIV- women	1 (0.4)	1	1	
HIV+ MSM	34 (13.6)	**38.5 (5.22–284.1)**	**59.0 (7.54–469.7)**	<0.001

SD, standard deviation; IQR, interquartile range; BMI, body mass index (Kg/m^2^); LSP, lifetime sexual partners; OR, odds ratio; 95% CI, 95% confidence interval. Bold values mean statistically significant.

MSM, Men having sex with men.

†Variables are adjusted for age, age at first sexual contact, LSP, marital/partnership status, and number of multiple infections. ‡*p*-value for adjusted OR, §HPV 16, 18, 6, 11, 31, 33, 45, 52, 58, §§HPV 16, 18, 6, 11, 31, 33, 45, 52, 58.

A crude and multivariable-adjusted logistic regression analysis revealed that the proportion of HPV infections containing one or more of the hrHPV genotypes was slightly higher among the cervical samples, whereas HPV infections containing one or more of lrHPV genotypes dominated significantly in the anal mucosa of MSM. This trend persisted when we restricted the analysis to those individuals harboring only-hrHPV or only-lrHPV genotype infections (Table 2). A closer look into genotypes of high clinical significance revealed that HPV 6, responsible for the majority of AGWs, was clearly and significantly predominant in the anal swab samples of HIV+ MSM [OR: 8.92 (3.84–20.7)], whereas the distribution of HPV 16, the most common carcinogenic genotypes, was not significantly different between the two sites [OR: 0.98 (0.59–1.61)].

**Figure 1:**
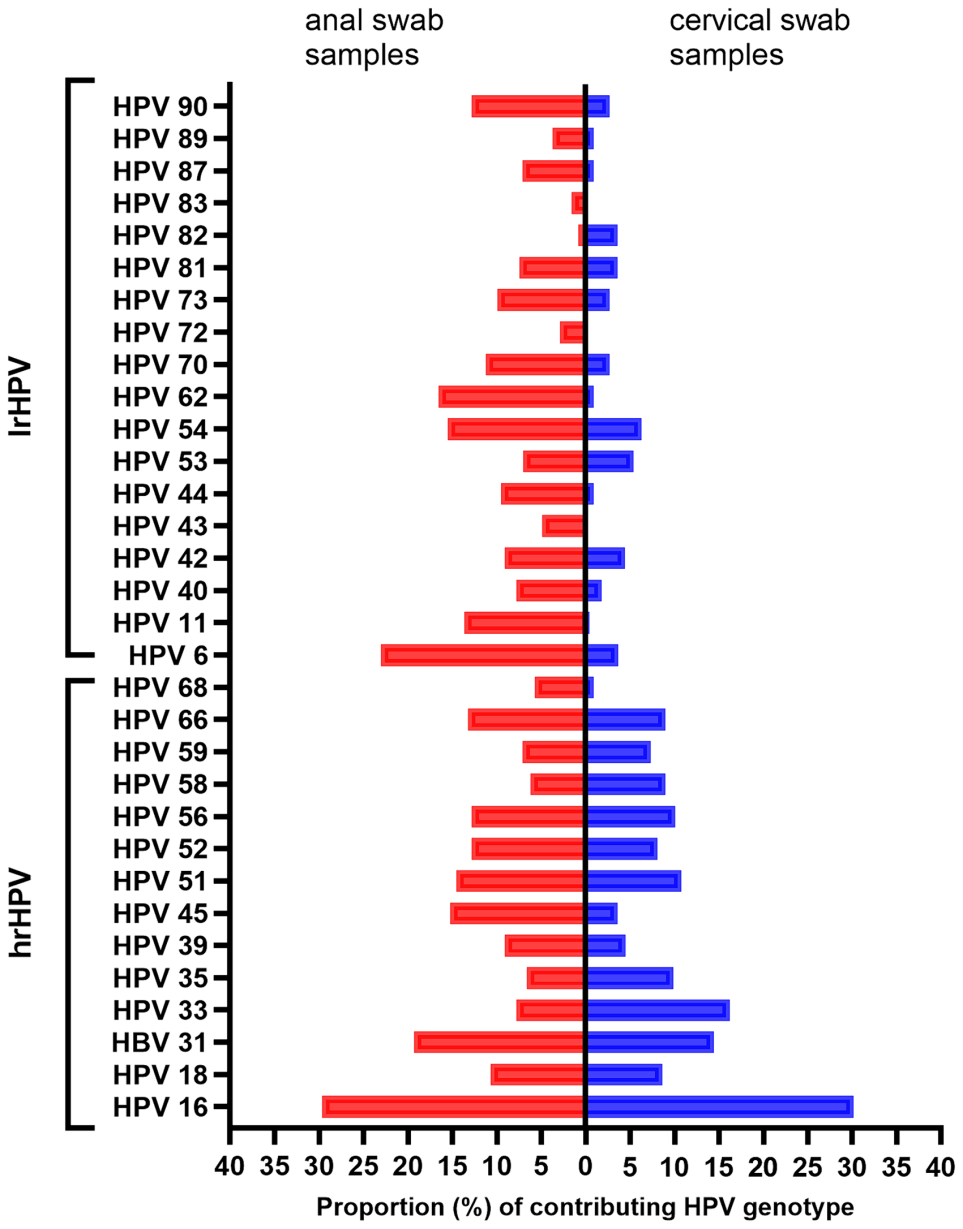
Distribution of HPV genotypes detected among HPV-positive adults. The red bars represent MSM and the blue bars represent women. lrHPV, low-risk HPV; hrHPV, high-risk HPV; MSM, men who have sex with men.

Figure 1 shows the proportional distribution of all detected hrHPV (16, 18, 31, 33, 35, 39, 45, 51, 52, 56, 58, 59, 66, and 68) and lrHPV (6, 11, 40, 42, 43, 44, 53, 54, 62, 70, 72, 73, 81, 82, 83, 87, 89, and 90) genotypes.

### 3.3 Restricting the analysis to single-genotype infections (*n* = 153)

We also characterized HPV infection patterns among those study participants positive for a single HPV genotype by excluding all infections containing two or more genotypes. An initial descriptive analysis showed that, overall, hrHPV infections are more commonly detected than lrHPV genotypes [65.5% (CI 58.6–73.0) vs. 34.5% (26.8–42.5)]. As shown in Table 3, the statistically significant discrepancy in the distribution of lrHPV and hrHPV between the two study populations persisted once again, strengthening our observation of the high tropism of lrHPV genotypes to the anal mucosa of MSM.

**Table 3:** Characterizing *single*-genotype HPV infections among HIV + MSM (*n* = 41) and healthy women (*n* = 118).

n (%)		Adjusted‡ OR (95% CI)	*p*-value
HR-HPV			
Healthy women	91 (77.1)	1	
HIV + MSM	14 (36.6)	**0.14 (0.06–0.28)**	**<0.001**
LR-HPV			
Healthy women	26 (22.0)	**1**	
HIV + MSM	28 (68.1)	**6.25 (2.91–13.4)**	**<0.001**
Vaccine-type HPV†			
Healthy women	63 (53.4)	1	
HIV + MSM	16 (39.0)	0.51 (0.25–1.06)	0.07
HPV 16			
Healthy women	24 (21.4)	1	
HIV + MSM	3 (7.1)	0.41 (0.05–3.73)	0.43
HPV 18			
Healthy women	5 (4.5)	1	
HIV + MSM	0 (0)	–	
HPV 6			
Healthy women	4 (3.6)	1	
HIV + MSM	3 (7.1)	2.08 (0.45–9.70)	0.34
HPV 11			
Healthy women	0 (0)	1	
HIV + MSM	2 (4.8)	–	

OR, odds ratio; 95% CI, 95% confidence interval; MSM, men having sex with men. Bold values mean statistically significant.

^†^HPV 16, 18, 6, 11, 31, 33, 45, 52, 58, ^‡^adjusted for number of lifetime sexual partners (*n* = 137).

### 3.4 Restricting the analysis to subjects with less than 10 LSPs (*n* = 135)

In order to account for the effect of high-risk sexual behavior on the observed trend, we conducted our analysis by restricting the data only to those participants reporting less than 10 LSPs. As shown in Table 4, the statistically significant discrepancies in the predominance of multiple-genotype, lrHPV, and hrHPV infections among the study population persisted despite the marked reduction in the sample size. HIV+ MSM (*n* = 48) were significantly more likely to harbor multiple-genotype and lrHPV infections (*p* < 0.001), whereas the hrHPV genotypes predominated significantly among healthy screening women (*p* < 0.001).

**Table 4:** Characterizing HPV infections among HIV + MSM (*n* = 48) and healthy women (*n* = 87) reporting <10 lifetime sexual partners.

n (%)		Adjusted‡ OR (95% CI)	*p*-value
Multiple HPV types			
Healthy women	43 (49.4)	1	
HIV + MSM	40 (83.3)	**11.5 (3.79–34.7)**	**<0.001**
HR-HPV			
Healthy women	79 (92.9)	1	
HIV + MSM	42 (87.5)	0.20 (0.04–1.06)	0.35
LR-HPV			
Healthy women	31 (35.6)	1	
HIV + MSM	41 (87.2)	**7.57 (1.31–43.8)**	**<0.001**
Vaccine-type HPV†			
Healthy women	63 (74.1)	1	
HIV + MSM	34 (70.8)	1.28 (0.73–2.25)	0.38
HPV 16			
Healthy women	28 (32.2)	1	
HIV + MSM	17 (35.4)	0.90 (0.35–2.29)	0.82
HPV 18			
Healthy women	5 (5.7)	1	
HIV + MSM	2 (4.2)	0.54 (0.07–4.19)	0.55
HPV 6			
Healthy women	3 (3.7)	1	
HIV + MSM	10 (20.8)	**9.62 (1.87–49.4)**	**0.007**
HPV 11			
Healthy women	0 (0)	1	
HIV + MSM	5 (10.6)	–	

OR, odds ratio; 95% CI, 95% confidence interval; MSM, men having sex with men. Bold values mean statistically significant.

^†HPV 16, 18, 6, 11, 31, 33, 45, 52, 58, ‡adjusted forage and the presence of multiple infections.^

## 4 Discussion

Our study presented a striking difference in the distribution of HPV genotypes across two population groups with independent circles of sexual practice. Although it may be considered a limitation to compare two different anatomical sites in two different population groups, this approach is justified by the fact that the examined anatomical areas are the main sites of receptive sexual contact for the respective population where the risk of microtrauma and hence HPV acquisition is the highest.

Our main finding was the discrepantly higher predominance of lrHPV genotypes, responsible for approximately 90% of all AGW cases, in the anal mucosa of HIV + MSM compared to cervical samples of healthy women. The relative distribution of hrHPV genotypes, on the other hand, showed a comparable presence between the two study populations with a slight predominance among women. The authors emphasize once again the fact that in this study, only HPV-positive adults were included, highlighting the pattern of genotype distribution once being positive for HPV. As described in the Methods section, the probability of being HPV positive is much higher among HIV+ MSM (244 out of 286 HPV-tested individuals) than among screening women (245 out of 1,320 HPV-tested women). Hence, the comparable distribution of hrHPV in the two populations is only relative. With an overall HPV prevalence of 85.3% among HIV+ MSM compared to 18.6% among screening women, the former group is at much greater risk of acquiring any relevant hrHPV and lrHPV.

The multi-partner nature of the sexual behavior among the HIV+ MSM in our study may be one plausible reason for the bulk of multiple-genotype infections, including lrHPV infection, in this group. However, the observed pattern in the analysis persisted even after adjusting for the number of genotypes per sample or even restricting the analysis to those individuals infected with single-genotype HPV infections, suggesting a genuine predilection of lrHPV genotypes for anal mucosa of this population. Furthermore, the restriction of the analysis to those participants reporting less than 10 LSPs did not blur the observed magnitude that genotypes highly linked to AGW circulate predominantly among MSM. The results from previous studies, including a large meta-analysis on anal HPV genotype distributions among MSM, women, and MSW, were similar to ours (21, 22). According to Giuliano et al., the prevalence of anal lrHPV vs. hrHPV was 33.3% vs. 29.1% among MSM, 9% vs. 5.5% among MSW, and 17.1% vs. 13.1% among women, supporting the notion of genuine predilection of lrHPV to this anatomical site (21). Similar distributions were observed elsewhere (23, 24).

A potential biological mechanism explaining this pattern of HPV infection may be the high diversity of mucosal microbiota in the anorectal tract compared to cervical mucosa, presumably supporting high rates of overall HPV positivity in the anal mucosa (25, 26). However, does a distinct microbiota composition explain the differential predominance of lrHPV? This speculation is supported by a previous study, which reported the explicit predominance of lrHPV genotypes among women with a highly diverse and peculiar microbiota composition in the vaginal mucosa (27). Zhou et al. showed that women with lrHPV infection exhibit a highly dysbiotic vaginal environment predominated by anaerobic bacteria with a significant reduction in the level of Lactobacilli compared to women with no lrHPV infection. Another study, which is based on the detection of HPV genotypes on placental swab samples, also supported the notion that not just HPV positivity but also the explicit presence of lrHPV is linked to distinct microbiota composition at the infection site (28).

Although the anus shares some anatomical/histological similarities with the cervix (29), the microanatomy of the anal transitional zone is not a one-to-one analogy of that of the cervix (30). According to Yang et al., the cells making up the anal transitional zone are arranged in multilayers in contrast to the cervical transitional zone, which is composed of a single layer of basal cells. This may be speculatively interpreted as better immunosurveillance and probably an earlier and more efficient clearance of a cervical infection compared to an anal HPV infection. Does this immunosurveillance function preferentially better for the clearance of hrHPV infection compared to lrHPV? In support of this speculation is the finding from a previous study among women with anal HPV infections, which reported a faster clearance of hrHPV genotypes such as HPV 16 compared to lrHPV from this anatomical site (22). On the contrary, the reverse is shown to be the case when it comes to cervical infections, where hrHPV genotypes take longer to be cleared compared to lrHPV infections (3, 31). Future studies that compare the humoral and cellular immunity distinctly post-hrHPV vs. lrHPV genotypes after natural infections may shed light on the potential role of the differential immune response as the main driving force.

One major limitation of this study is the fact that the MSM in our study population are HIV-positive, and we lack comparable data on HPV genotype distribution among HIV-negative MSM. Although HIV-negative MSM have a lower prevalence of HPV infection compared to HIV+ ones, epidemiological studies suggest that lrHPV genotypes are commonly detected in the anal mucosa irrespective of the HIV status (21, 32). Similarly, data on cervical HPV genotype distribution among HIV+ women might have enhanced the comparability of our study population. Although previous studies, including meta-analyses, showed an increased overall risk of cervical HPV positivity among HIV+ women compared to HIV-negative ones, none of these studies suggested a higher proportion of lrHPV genotypes within HPV-infected women (32, 33). These findings may be suggestive of a lesser role of HIV positivity as a risk factor for the observed predominance of lrHPV genotypes among HIV+ MSM. However, further studies with comparable populations are recommendable.

A further limitation is the fact that the sample size was too small to conduct any meaningful single-genotype analysis, for example for HPV6 or HPV 11. Although single-genotype infections are rare in the MSM population, a study with a much larger sample size may achieve this goal. The fact that the HPV detection kit used was diagnostically approved for the analyses of clinical samples such as cervical, vaginal, urethral, buccal swabs, seminal fluid, and vaginal secretions but not explicitly for anal samples may be a limitation. However, by using positive and negative controls as well as by amplifying the housekeeping gene beta-globin, for each of the samples used, we were able to ascertain the soundness of the method on anal samples as well.

The observation that lrHPV genotypes preferentially strike the anal mucosa of a predisposed population is of high clinical significance. HPV 6 and 11, in particular, showed obvious predilection to the anal mucosa, strengthening further the case for a targeted HPV immunization of MSM to curtail the burden of anal warts in this population. Although benign, AGWs are known to recur despite repeated and invasive therapy, putting the affected in enormous physical and emotional distress (34). Although designated as lrHPV, there exists tangible evidence that these genotypes may be associated with malignant transformations as well (35–39). Laser capture microdissection assays have proven that anal cancer lesions occasionally contain only lrHPV genotypes, suggesting a possible causal role of these genotypes in the pathogenesis of invasive lesions (36). Chronic inflammatory damage due to recurrent warts may be a predisposing factor either through increased risk of mutations or through increased predisposition to infections with other HPV types like the oncogenic ones (40–42). It was also reported that the extent of lrHPV-associated squamous cell carcinomas is much higher in the anal canal than in cervical cancer (7).

These observations suggest that our finding of disproportionately high predominance of lrHPV is of non-negligible clinical and public health importance, deserving serious attention with respect to preventive strategies. Although the free-of-charge HPV immunization program is designed in a gender-neutral manner in several countries, such as Austria, the low coverage rate and poor vaccine acceptability—particularly among boys/men (43)—make it a point to modify this program to include non-vaccinated MSM, particularly those with HIV infection, in this vaccination program.

## Data Availability

The raw data supporting the conclusions of this article will be made available by the authors, without undue reservation.

## Supplementary material

SUPPLEMENTARY FIGURE 1 Overview of the source- and the study-population.

SUPPLEMENTARY FIGURE 2 Characteristics of HPV infection among the source population (cervical samples of *n* = 1,320 women and anal swab samples of *n* = 286 MSM). Presented are the overall, multigenotype, high and low-risk HPV genotypes with a 95% CI. 95% CI is calculated using the program Epitools (https://epitools.ausvet.com.au/ciproportion) using the Wilson method. HPV, human papillomavirus; MSM, men who have sex with men.
