# Supplementary figures and images for: Disproportionate preponderance of HPV genotypes associated with anogenital warts among HIV-positive MSM
Source: Front Public Health. 2024 Sep 20;12:1437309. doi: 10.3389/fpubh.2024.1437309 (PMC11449850; doi:10.3389/fpubh.2024.1437309)

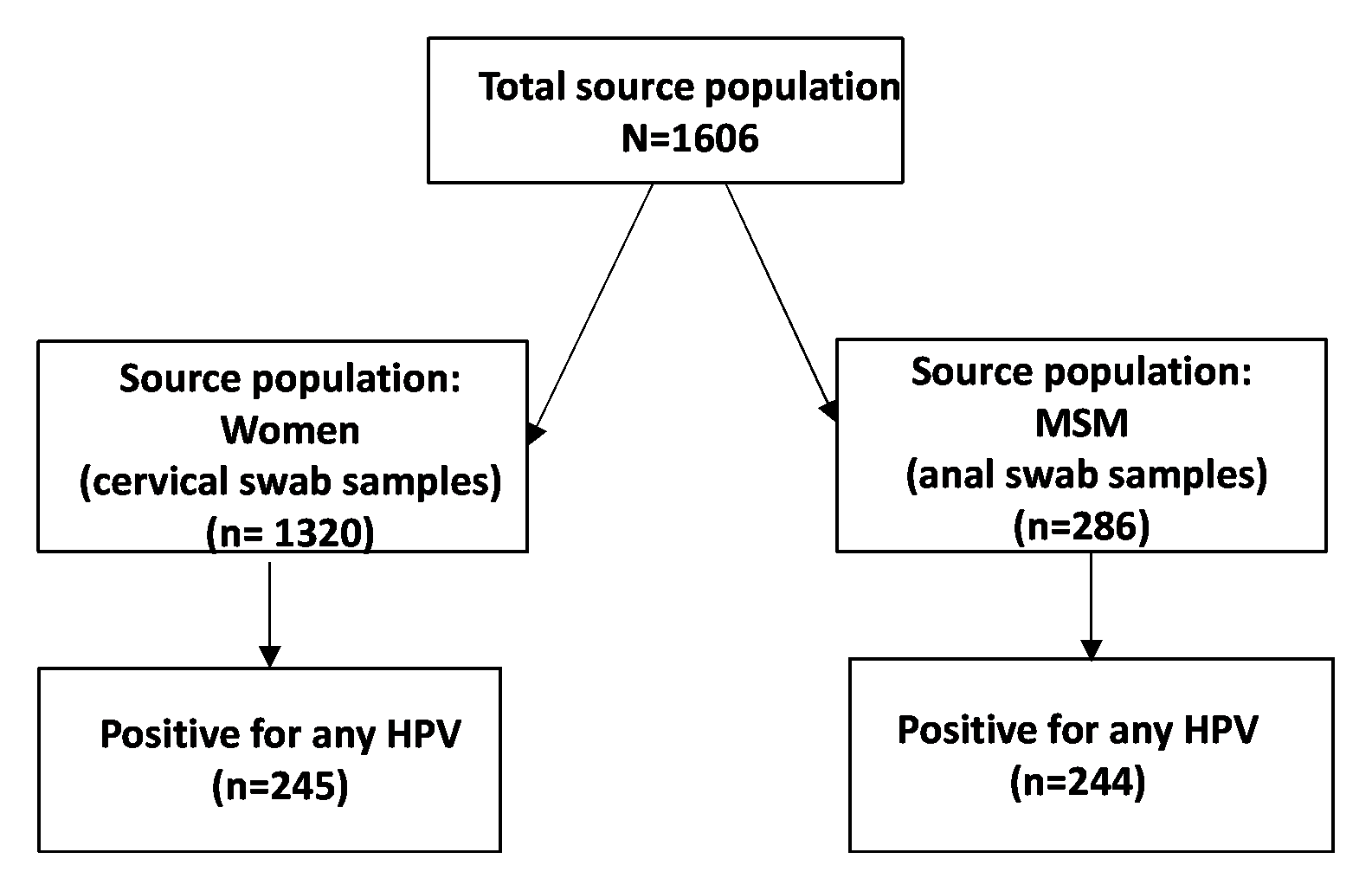

Supplement: SUPPLEMENTARY FIGURE 1 — Overview of the source- and the study-population. [file Image_1.PNG]

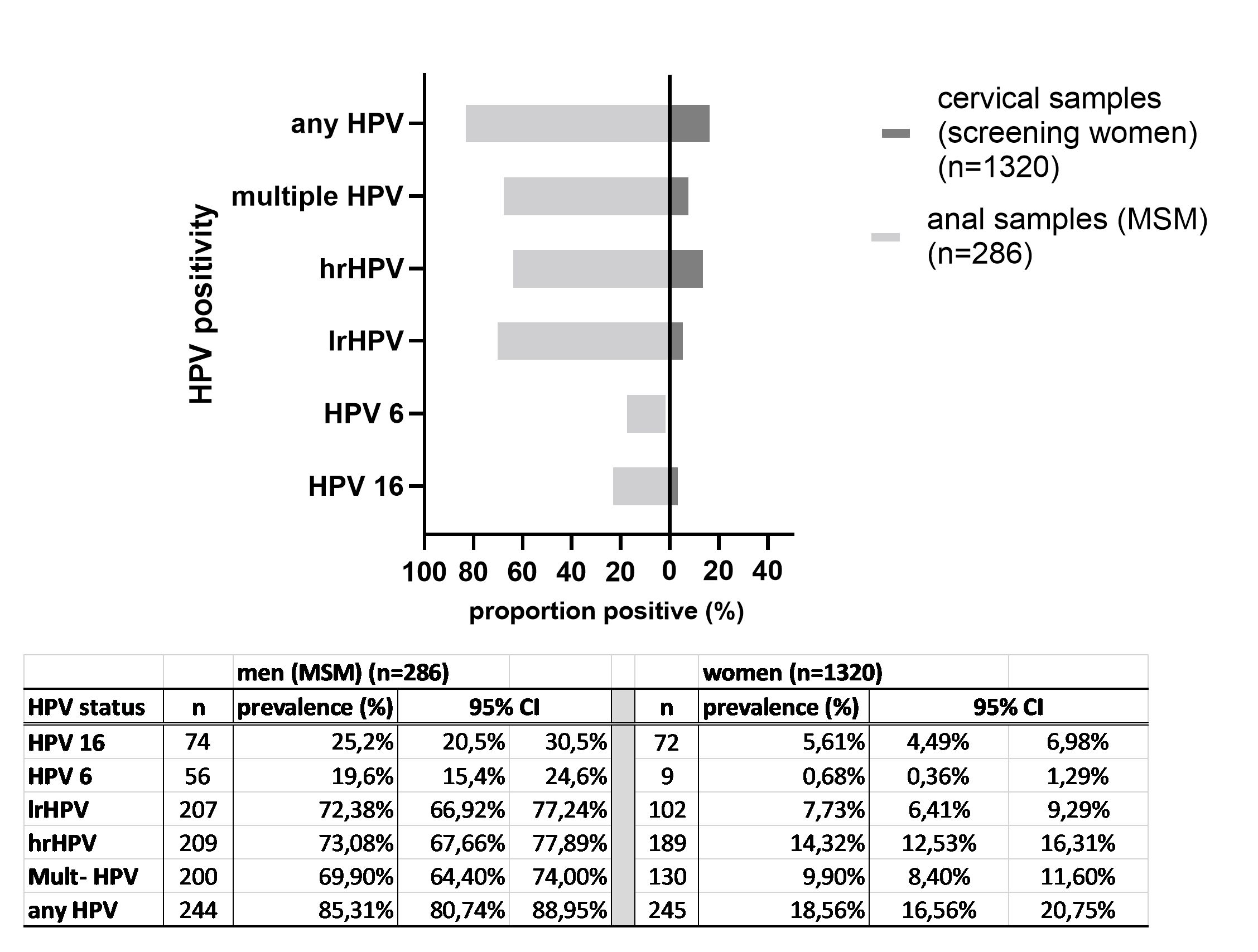

Supplement: SUPPLEMENTARY FIGURE 2 — Characteristics of HPV infection among the source population (cervical samples of n = 1,320 women and anal swab samples of n = 286 MSM). Presented are the overall, multigenotype, high and low-risk HPV genotypes with a 95% CI. 95% CI is calculated using the program Epitools (https://epitools.ausvet.com.au/ciproportion) using the Wilson method. HPV, human papillomavirus; MSM, men who have sex with men. [file Image_2.PNG]
